# Monkeypox: A New Threat?

**DOI:** 10.3390/ijms23147866

**Published:** 2022-07-17

**Authors:** Dorota Kmiec, Frank Kirchhoff

**Affiliations:** Institute of Molecular Virology, Ulm University Medical Center, 89081 Ulm, Germany; frank.kirchhoff@uni-ulm.de

**Keywords:** monkeypox, MPXV, zoonosis, emerging viruses, 2022 MPXV outbreak

## Abstract

The global vaccination programme against smallpox led to its successful eradication and averted millions of deaths. Monkeypox virus (MPXV) is a close relative of the Variola (smallpox) virus. Due to antigenic similarity, smallpox vaccines cross-protect against MPXV. However, over 70% of people living today were never vaccinated against smallpox. Symptoms of monkeypox (MPX) include fever, head- and muscle ache, lymphadenopathy and a characteristic rash that develops into papules, vesicles and pustules which eventually scab over and heal. MPX is less often fatal (case fatality rates range from <1% to up to 11%) than smallpox (up to 30%). MPXV is endemic in sub-Saharan Africa, infecting wild animals and causing zoonotic outbreaks. Exotic animal trade and international travel, combined with the increasing susceptibility of the human population due to halted vaccination, facilitated the spread of MPXV to new areas. The ongoing outbreak, with >10,000 cases in >50 countries between May and July 2022, shows that MPXV can significantly spread between people and may thus become a serious threat to public health with global consequences. Here, we summarize the current knowledge about this re-emerging virus, discuss available strategies to limit its spread and pathogenicity and evaluate its risk to the human population.

## 1. Monkeypox Discovery and Outbreaks

**Figure 1:**
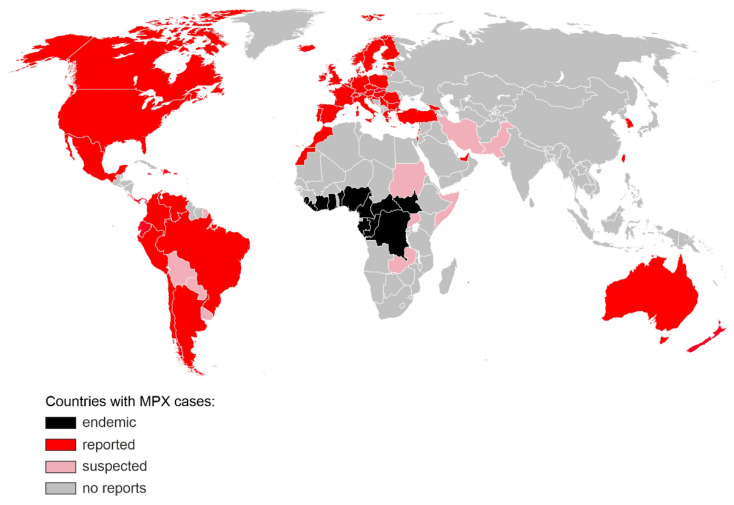
Countries with confirmed (red) or suspected (pink) MPX cases during the 2022 non-endemic outbreak. Regions, where MPX was endemic prior to 2022, are shown in black. The map includes cases reported until 12 July 2022 [8]. The base layer map was obtained from https://commons.wikimedia.org/wiki/File:BlankMap-World.svg (accessed on 1 July 2022).

Monkeypox virus (MPXV) was identified in 1958 during outbreaks of a pox-like disease in macaque monkeys kept at a research facility in Denmark, hence the disease name “monkeypox” (MPX) [1]. The first human case was discovered in 1970 in the Democratic Republic of the Congo (DRC) during intensified smallpox surveillance [2]. The patient, a 9-months-old baby, developed tell-tale signs of MPX including fever and a pox-like rash which developed into haemorrhagic lesions that crusted over and healed over the next 2 weeks. Despite the initial recovery, the patient succumbed to secondary infections and died in the hospital. Within the next decade, additional MPX cases, mainly among children (83% of all cases), have been identified in the Democratic Republic of the Congo (DRC, formerly Zaire from 1971 to 1997) as well as four additional Central and West African countries: Liberia, Sierra Leone, Nigeria and Ivory Coast [3]. In the 1970s and 80s, most of the reported cases occurred in DRC, with an estimated 11% case fatality rate (CFR) among those who had not received a smallpox vaccination. The CFR was the highest among children under 4 years (15%) [4]. Extended surveillance led to the identification of additional MPX-endemic regions in Benin, Cameroon, the Central African Republic, Gabon, Ghana, Sierra Leone, as well as South Sudan and confirmed that most of the cases occurred in the DRC [5]. The first outbreak of MPXV in humans outside of Africa was documented in 2003 in the United States and linked to an exotic pet import from Ghana. In total, 71 MPX cases but no deaths were reported, all in patients who had been exposed to infected prairie dogs [6]. In recent years, a few small clusters and single MPX cases were identified in the UK (2018 and 2019), Israel (2018), Singapore (2019) and the US (2021), all linked to travel to Nigeria, which has experienced re-emergence of MPX and reported over 500 suspected cases since 2017 [7]. On the 4 May 2022, a patient with a recent travel history to Nigeria and an unexplained rash presented to a UK hospital. Polymerase chain reaction (PCR) on a vesicular swab confirmed a MPX diagnosis [5]. Within the next months, thousands more cases were identified in over 50 countries on 6 continents, with major clusters in England, Germany, Spain, France and Portugal [8] (Figure 1).

This is the largest and most dispersed non-endemic MPXV outbreak known to date. Unlike previous outbreaks, there is no clear link between the infected people or a shared virus exposure source, such as travel to an endemic region or handling infected animals. While the transmission dynamics and route of infection are still uncertain, the virus appears to spread through close physical contact, and the vast majority of the affected are young or middle-aged men who have sex with men (MSM) and had recent sexual contact with new or multiple partners [5]. Diagnosed patients are being advised to isolate and their close contacts traced. As of 12 July 2022, no MPX-associated fatalities have been reported in the non-endemic areas. In the light of a rapid increase in cases, and unprecedented scale of human-to-human transmission, WHO increased the risk level to global public health of MPX from low to moderate, with a high risk level in the European Region which accounts for over 80% of all new MPXV infections [5].

## 2. Virus Classification and Phylogeny

**Figure 2:**
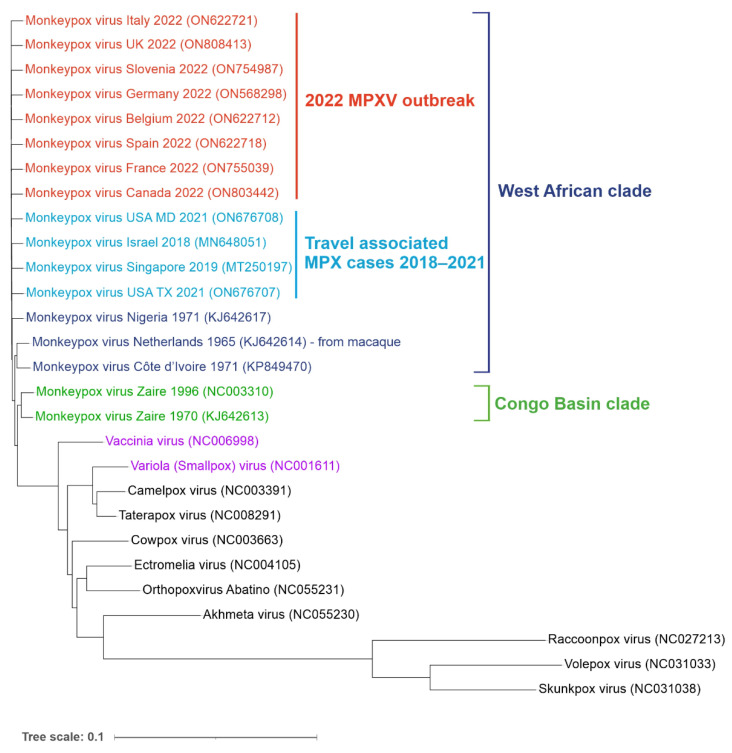
Phylogenetic tree of orthopoxvirus genomes. Reference genomes and MPXV genomes from the non-endemic 2022 outbreak (shown in red) as well as travel-associated cases detected in recent years (light blue). West African (dark blue) and Congo Basin (green) clades are indicated. Vaccinia (VACV) and Variola (VARV) viruses are shown in purple. Aligned sequences were obtained from the NCBI virus database. Genbank accession numbers are shown in the brackets. The tree was generated using ngphylogeny.fr and visualized using iTOL online tools (itol.embl.de/) (both accessed on 1 July 2022).

MPXV is a double-stranded DNA virus belonging to the Orthopox genus in the family Poxviridae. Its closest known relatives include Variola (VARV) and Vaccinia viruses (VACV) (Figure 2).

The genomes of MPXV and VARV share a high level of sequence similarity (96%); however, phylogenetic studies suggest that they did not evolve from one another. VARV is more closely related to the Camelpox virus and the Taterapox virus found in gerbils (98% genome similarity) and both VARV and MPXV may have originated from poxviruses infecting rodents and/or ruminants [9,10,11,12]. MPXV strains are grouped into two clades: Congo Basin (CB) and West African (WA). The more pathogenic CB clade (CFR up to 11%) is found mainly in the DRC and surrounding counties and was responsible for the first documented human case of MPX in 1970 [2]. The ongoing 2022 outbreak, as well as the previous smaller outbreaks in Texas (2003), the United Kingdom (2018 and 2021), Singapore (2019) and Israel (2018–2019), were all caused by the WA clade characterized by lower CFR (estimated between less than 1% to 3.6%) [13,14,15,16,17]. Phylogenetic analysis of sequenced MPXV genomes from the current outbreak revealed two distinct virus lineages. Almost all strains from the 2022 non-endemic outbreak form a single subclade (B.1) and most likely have a single origin [18]. This lineage is most related to a single case in a traveller from Nigeria to Maryland in 2021. The second lineage (A.2) currently includes only two strains identified in the US, which differ by 80 nucleotide changes relative to the other 2022 MPXV sequences, suggesting an independent virus introduction event [19].

## 3. Animal Reservoir

MPXV has been detected in various species, and it is still not clear which of them serves as the main animal reservoir. For example, the virus has been isolated from African squirrels and MPXV reactive antibodies or viral DNA have been detected in multiple wild rodent and shrew species as well as pigs [20,21,22,23,24,25]. The largest documented series of zoonotic MPXV transmissions occurred in 2003 in the US, when 71 people became infected after handling prairie dogs. These pets had been housed together with infected dormice, rope squirrels and a Gambian giant rat imported from Ghana [26]. There is also evidence of non-human primate infections in the wild. For example, MPXV has been isolated from a dead sooty mangabey monkey [27], and poxvirus-reactive antibodies have been detected in 2% of Zambian baboons as well as Cercopithecus and Colobus monkeys [28]. A MPXV outbreak has been reported in a population of chimpanzees, with symptoms resembling those of humans [29]. In addition, a case of MPXV infection in a baby bitten by a wild chimpanzee was described [30]. Apart from contact with live animals, preparation or consumption of wild game or bushmeat also causes a risk of contracting MPXV. However, it is frequently impossible to establish the exact zoonotic source and route of transmission in endemic regions due to concurrent exposure to multiple wild species [5,31]. Thus, unlike VARV which is confined to humans, multiple mammalian species could serve as natural animal reservoirs of MPXV, which poses a significant challenge to MPXV control and containment efforts.

## 4. Virus Genome and Morphology

**Figure 3:**
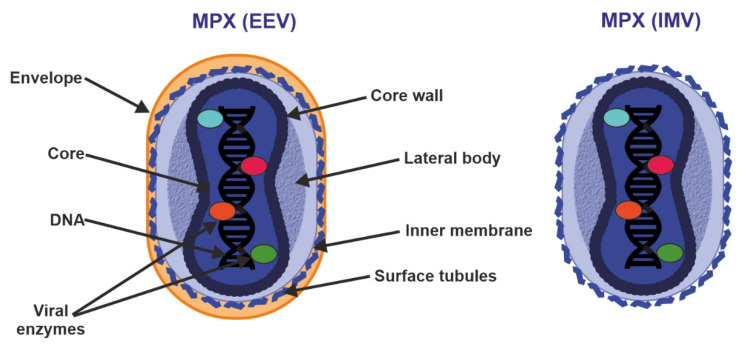
Structure of enveloped extracellular virions (EEV) and intracellular mature virions (IMV). See text for further details.

The MPXV genome encompasses ~197,000 bp and includes hairpin termini as well as >190 non-overlapping open reading frames (ORFs) [10,32]. The highly conserved central coding region of the genome is flanked by variable ends that contain inverted terminal repeats. At least 90 ORFs are known to be essential for poxvirus replication and morphogenesis. Many of the additional so-called non-essential ORFs play a role in the differences in poxvirus host tropism, immunomodulation and pathogenesis, with many ORFs still waiting to be functionally characterized [33]. MPXV virions are barrel- or oval-shaped particles, with an average size of ~280 nm × 220 nm [16]. Mature poxvirus particles have a characteristic dumbbell-shaped nucleoprotein core containing a large double-stranded linear DNA genome [6]. Similarly to VACV, MPXV virions contain over 30 structural and membrane viral proteins as well as virus-encoded DNA-dependent RNA polymerase and associated transcriptional enzymes [34,35] (Figure 3).

Poxvirus particles have two mature forms, both of which can mediate infection: extracellular enveloped virus (EEV) (thought to be responsible for early dissemination) and the intracellular mature virus (IMV) released during cell lysis [36,37]. The major structural difference between IMV and EEV is that IMVs lack the additional outermost membrane layer. However, the levels of incorporated viral proteins also differ between the two types of virions [35,36].

## 5. Transmission and Replication Cycle

**Figure 4:**
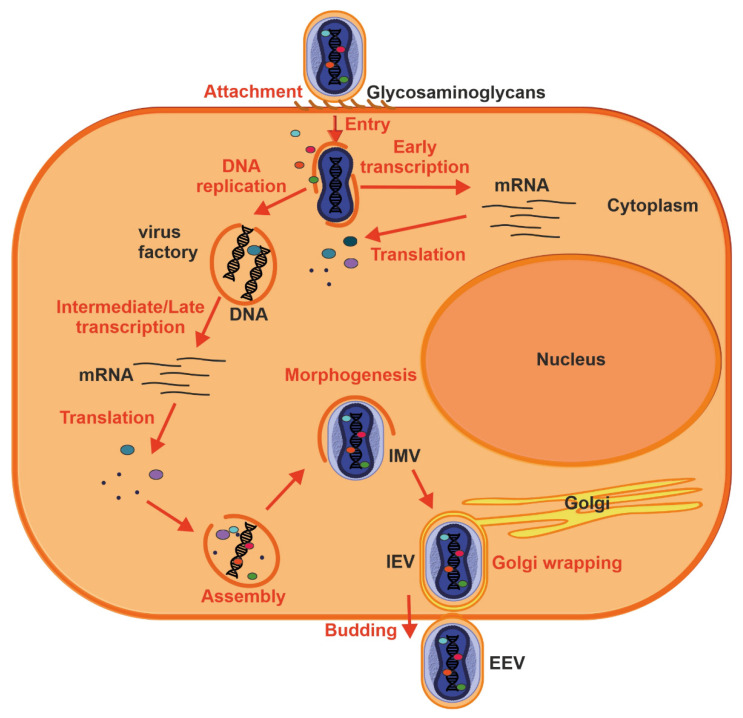
Replication cycle of a poxvirus. Key events are outlined: attachment (1), entry (2), early viral gene transcription and translation (3), DNA replication (4), intermediate and late transcription and translation (5), assembly (6), morphogenesis (7), envelopment by intracellular membranes (8) and budding (10). See text for further details.

Handling infected rodents appears to be a common source of zoonotic MPXV transmission, and human-to-human spread can occur through close contact with lesions, body fluids, respiratory droplets and contaminated objects [5]. Studies using macaques that were exposed to aerosolized MPXV showed that the pathogen initially infects lower airway epithelial cells and spreads to lymph nodes, followed by systemic dissemination through monocytic cells. MPX lesions may subsequently form in lymph nodes, thymus, spleen, skin, oral mucosa, gastrointestinal tract and reproductive system [38]. In vitro studies suggest that the MPXV can infect most mammalian cells [39,40]. Widely abundant glycosaminoglycans such as chondroitin and heparin sulfates, as well as laminin, play a role in cellular attachment of other poxviruses [41,42,43] (Figure 4).

Proteins involved in glycosaminoglycan biosynthesis were also recently identified in a genome-wide screen for factors facilitating MPXV infection [44]. Thus, MPX virion attachment is most likely mediated by external virion proteins and cellular glycosaminoglycans on the surface of the target cell or by components of the extracellular matrix. Following attachment, poxviruses enter the host cells by a low pH endosomal pathway or direct fusion with the plasma membrane at neutral pH, which releases the viral core in the cytoplasm. Fusion of IMV and EEVs with the cell is dependent on a complex of ~12 non-glycosylated, viral membrane proteins [45]. Following entry, viral transcription is initiated by the virus-encoded multi-subunit DNA-dependent RNA polymerase followed by the translation of early, intermediate and late proteins on host ribosomes [46]. Poxvirus DNA synthesis occurs in cytoplasmic structures, often referred to as “factories”, which gradually transition from compact DNA-containing structures wrapped by ER membrane to crescent-shaped structures where virion assembly occurs [16,47]. While the majority of mature virions remain inside of the cell (IMV), some are transported via microtubules and become enveloped by two ER or Golgi-derived membranes. These enveloped virions can initiate actin polymerization, which propels the particle on an actin tail toward an adjacent cell, or exit the cell by fusion with the cytoplasmic membrane and become EEV [47].

## 6. Immune Evasion

Poxviruses use multiple mechanisms to evade the recognition and targeting by the immune system of their hosts. In vitro, MPXV infection of human cells does not induce interferon-stimulated gene (ISG) expression and further suppresses Tumour Necrosis Factor alpha (TNF-α), Interleukin 1 alpha and beta (IL-1α and β), C-C Motif Chemokine Ligand 5 (CCL5) and Interleukin 6 (IL-6) activation in primary fibroblasts [48]. MPXV infection leads to the accumulation of less dsRNA than the VACV and prevents phosphorylation of pattern recognition immune receptor Protein kinase R (PKR) and Eukaryotic Initiation Factor 2 alpha (eIF2α), which suppresses the activation of antiviral responses [49]. The MPXV genome encodes multiple proteins that promote its immune evasion. For example, viral protein B16 inhibits antiviral type I interferon-induced signalling [50]. A homolog of D7L has been reported to inhibit the proinflammatory cytokine Interleukin 18 (IL-18), which plays a crucial role in the control of monkeypox viraemia in mice [51]. Zinc-finger antiviral protein (ZAP) selectively targets CpG dinucleotides in RNAs and exerts selective pressure against CpGs in viral genomes [52,53,54]. However, the MPXV genome and mRNAs are not specifically suppressed in CpGs. It was shown that the C16 protein of VACV sequesters ZAP and counteracts its antiviral activity [55], and its homolog in MPXV might have a similar role. Another example of an MPXV immunomodulator is the complement control protein (CCP), which prevents the initiation of the complement activation pathway [56]. The WA clade, which lacks the CCP gene, has a lower case fatality rate than the CB clade [57]. Removal of CCP from the CB MPXV strain reduced disease morbidity and mortality in prairie dogs [56]. However, additional, yet to be determined, factors contribute to the difference in WA and CB clade pathogenicity. MPXV also interferes with adaptive immune responses of antiviral CD4+ and CD8+ T cell responses by preventing T cell receptor-mediated T cell activation [58]. As many more MPXV ORFs remain to be functionally characterized, we are still far from a complete understanding of MPXV immunomodulators.

## 7. Mutation and Adaptation

DNA genomes such as those found in MPXV mutate much less frequently than those of RNA viruses. This is due to the higher stability of double-stranded DNA and the 3′–5′ proofreading exonuclease activity of poxvirus DNA polymerase [46]. The evolutionary rate of VARV was estimated at 1–2 nucleotide changes per year and this number is likely similar for MPXV [59]. The genomes of the first West African MPXV isolate from 1971 and strains from the 2022 MPXV outbreak differ by less than 0.06%. Nucleotide composition analysis of the MPXV genome revealed that its AT content is about two times higher than the GC content [18]. Mammalian DNA and RNA binding or editing enzymes are known to exert selective pressures on viral genomes, often introducing a bias in genomic nucleotide usage. For example, APOBECs can accelerate viral mutation rates, leading to a decrease in C content and an increase in T content due to cytosine deamination [60,61,62]. While early studies suggested that short-term VACV replication is not affected by APOBEC3 family members [63], analyses of MPXV genomes from recent years and the ongoing 2022 outbreak revealed that ~90% of new nucleotide changes were characteristic of the APOBEC3 editing [18,19]. Of 13 mutations differentiating MPXV genomes of the current outbreak from the closely related 2021 isolate, seven result in amino acid changes in proteins responsible for viral transcription, ss/dsDNA binding, entry/fusion complex formation, inhibition of IL-1/TLR signalling and EEV envelopment and egress [19]. Poxviruses may also gain or lose genes through recombination, retaining beneficial elements in response to selective pressures without excessive expansion of their genomes [64]. Indeed, a recombination event leading to a loss of ∼10,000 bp genome fragments from MPXV in West Africa was associated with the divergence of the two MPXV clades [56,65]. Recent microevolution in the 2022 MPXV genomes resulted in a subset of strains carrying a 913 bp frameshift deletion in the homolog of VACV Ankyrin/Host Range D7L protein responsible for IL-18-binding and immune evasion [18,51]. The functional impact of the unique mutations found in the 2022 outbreak strains is still unknown.

## 8. Pathogenesis and Clinical Features

Typical symptoms of MPX include head- and body aches, fever, chills, sore throat, malaise, fatigue, enlarged lymph nodes and a characteristic skin rash that develops into papules and vesicles which eventually crust over and heal. The disease manifestation is very similar to chickenpox and may hence be misdiagnosed, although lymphadenopathy is more common in MPXV infections [4,66]. However, in the current 2022 outbreak, many patients show atypical disease presentation, including no or few lesions, which are often localized in the genital or perineal/perianal area, anal pain and bleeding [67]. The onset of MPX symptoms ranges from 5 to 21 days, and typically the infection resolves on its own within 2–4 weeks [68]. MPXV infection can cause prolonged viral DNA shedding in the upper respiratory tract that persists after skin lesion resolution, but it is unclear if this is associated with infectious virus transmission [68]. The majority of MPXV infections resolve on their own. However, the disease is more severe and might require hospitalization in young children and immunosuppressed patients [4,69]. HIV-1 infected individuals have prolonged MPX illness, larger lesions, and higher rates of both secondary bacterial skin infections and genital ulcers [70]. Furthermore, during pregnancy, MPXV can be transmitted through the placenta and lead to foetal death [71]. In some cases, potentially life-threatening complications such as encephalitis, secondary infection of the integument, bronchopneumonia and sepsis can occur [4]. Another rare, but serious, long-term complication of MPX is the loss of vision resulting from infection of the eye cornea and tissue scarring [4]. The overall mortality rate varies depending on patient age, virus clade and localization of the outbreak. Similarly to smallpox, MPX is more often fatal in children than in adults [72,73]. MPX CB clade has case fatality rates of up to 10%, while for the WA clade it is around 3.6% [4,57,74]. However, these CFRs only apply to African outbreaks. MPX disease outcomes in the non-endemic regions were always favourable, possibly due to better healthcare access and treatment of secondary infections or pre-existing conditions. Since the beginning of 2022 at least 1536 suspected MPX cases and 72 deaths were reported in the endemic regions, mostly in DRC [5]. In contrast, in the 2003 US outbreak of MPXV that involved 71 cases no deaths were recorded, although two children became severely ill, one with encephalitis [75]. Similarly, as of 12 July 2022, no deaths have occurred in the ongoing 2022 outbreak outside of the endemic areas, although some patients required hospitalization [76].

## 9. Vaccination

Due to the antigenic similarity between VACV and MPXV, smallpox vaccination is considered one of the measures to control MPXV outbreaks [77]. Data collected between 1980 and 1984 show that the overall attack rate for unvaccinated MPX case contacts (7.2%) differed significantly from the attack rate for vaccinees (0.9%) [78]. The number of reported human MPX cases in DRC has increased by over 20-fold between the 1980s and 2007 and the median age of MPX patients increased, presumably due to the halted smallpox vaccination [79]. Population studies in DRC in the 1980s found that the vaccination imparted ~85% protection against MPX [80]. Data collected in DRC 2005–2007 suggest that, even after 25 years, vaccinated individuals have a significantly reduced risk of MPX [79]. Similar observations were made following the 2003 US MPXV outbreak when three asymptomatic cases were discovered in pre-immune individuals [26]. In the past, smallpox vaccines contained live unattenuated VACV strains. This type of vaccine is very effective due to its high immunogenicity but can cause adverse reactions in immunocompromised individuals. Furthermore, in a small number of cases, replication-competent VACV strains have been found to be transmitted to close contacts of vaccinees [81]. Currently, there are two types of smallpox vaccines approved for human use in the US and Europe. Single-dose ACAM2000 and Aventis Pasteur Smallpox Vaccine (APSV) contain replication-competent live vaccinia virus and cannot be used in the immunocompromised. The most recently approved two-dose Modified Vaccinia Ankara virus Bavarian Nordic (MVA-BN) vaccine (brand names JYNNEOS, IMVAMUNE, IMVANEX) contains a replication-deficient virus and is safe for the immunocompromised [82,83,84,85]. While it is still unknown if the replication-competent and non-replicating vaccines are equally effective in humans, MVA and MVA-BN vaccination was shown to offer full protection against severe MPX in non-human primates [82,83]. In addition, a highly attenuated smallpox vaccine LC16m8 with an improved safety profile has been developed and licensed in Japan [86]. While the available data support the notion that smallpox vaccination may offer long-term protection from MPX, more studies are needed to establish how long the protection lasts, especially in the vulnerable populations and the immunocompromised. US and UK were the first countries to pursue a ring-vaccination approach and offered pre- and post-exposure vaccination to close contacts of MPX cases in the 2003, 2018 and 2019 outbreaks [26,87]. In the last month, more and more countries decided to offer vaccination to people exposed to MPXV or at risk of acquiring it, however, the scale of the uptake is unknown. WHO’s interim guidance released on 14 June does not recommend mass vaccination against MPXV, except for recently exposed individuals, health workers providing care to MPX patients and laboratory personnel working with the virus or performing diagnostics on patient samples [88]. Despite this, in June 2022, The New York City Department of Health and Mental Hygiene and the UK Health Security Agency started offering the vaccine to gay and bisexual men at higher risk of exposure to help control the recent outbreak of the virus [89,90]. Since then, many other regions with high numbers of new cases started doing the same, although the vaccine availability is still low. In June 2022, the European Health Emergency Preparedness and Response Authority (HERA) ordered 110,000 doses of non-replicating Modified Vaccinia Ankara Bavarian Nordic (MVA-BN) vaccine in response to the current MPXV outbreak.

## 10. Treatment

Due to the relatively long incubation period of MPX (5 to 21 days), early post-exposure vaccination can offer benefits to vulnerable patients. A decrease in the severity of MPX disease after post-exposure vaccination was demonstrated by challenge studies of prairie dogs [91] but no clear benefit was reported in studies on non-human primates [92,93]. These differences may be due to an extended incubation period of the virus in the intranasal prairie dog infection model, which better reflects the course of a typical human infection and allows sufficient time for mounting an immune response [94]. Passive vaccination with immune sera or vaccinia immune globulin have been used in close contacts of smallpox cases and immunocompromised patients with progressive vaccinia [95,96]. Although no clinical trial data on the efficacy of vaccinia immune globulin intravenous (VIGIV) against MPX are available, the therapy is safe and could be beneficial in the treatment of vulnerable patients. Tecovirimat (ST-246) is the only FDA and EMA-approved drug for orthopoxvirus infection in humans. It is not widely used but has been applied in 2021 to treat severe MPX in patients, who eventually made a recovery [97]. Tecoviromat interferes with the cellular localization of the p37 viral envelope protein, preventing its membrane trafficking and inhibiting the formation of enveloped virions and their dissemination [98]. This drug was shown to prevent MPX mortality in the primate model without adverse effects [99,100]. In addition, cidofovir, a drug approved for cytomegalovirus (CMV) retinitis treatment in AIDS patients, was shown to be effective against lethal MPXV infection in macaques [101]. However, there are no data on the effectiveness of cidofovir against MPX in patients. Since only limited data on the effectiveness of the approved drugs against MPX in humans are available, their use will likely remain limited to the treatment of severe cases.

## 11. Discussion

The rapid increase in MPX cases in non-endemic regions suggests that this zoonotic virus can efficiently spread between people and thus could pose a risk to global public health. Between 4 May and 12 July 2022, more than 10,000 new non-endemic MPX cases have been reported globally. This number is likely underestimated due to the limited access to MPXV diagnostics in many regions [5]. Disease surveillance will be a crucial factor, not only in the ongoing risk evaluation of MPX threat level but also in containing and controlling the outbreak. While further studies are urgently needed to establish if the currently circulating MPXV strains differ in their transmissibility and/or pathogenicity from previous isolates, host factors such as international travel and increasing poxvirus susceptibility facilitated the global spread of this re-emerging pathogen. The increase in MPX cases coincides with waning immunity due to the halted smallpox vaccinations, which cross-protected against MPX. During the 70s and 80s, endemic MPX cases were predominantly reported among unvaccinated children [4,80]. However, the median age of the MPX patients increased with time due to the susceptibility of new generations born in the post-smallpox eradication era [57]. While the evidence shows that smallpox vaccines effectively protect against MPX, the scale of the current outbreak is still too small to justify mass vaccination. Instead, offering the vaccine to certain groups, such as MSM at risk of exposure, healthcare personnel and close contacts of MPX patients is a promising strategy for containing the MPXV outbreak. Furthermore, drug and immunoglobulin-based treatment options offer hope that the worst disease outcomes can be prevented. Considering the low mutation rates of MPXV and long-lasting vaccine-induced immunity, effective escape of adaptive immune responses is unlikely, but cannot be completely excluded. Therefore, further studies on this re-emerging pathogen, especially the 2022 outbreak strains, are highly warranted.

In the current non-endemic outbreak, a new high-risk group has been identified—men who have sex with men (MSM) with recent contact with new/multiple sexual partners. This suggests that MPXV is being transmitted through close physical contact. Importantly, however, both the past and current MPXV outbreaks show that this virus can infect and be transmitted between individuals of any sex, age and orientation. The lack of reported MPX deaths in the non-endemic regions supports the notion that the clinical consequences of the current outbreak are less severe compared to those observed in endemic regions. However, the current demographic of MPX patients still includes very few cases among the most vulnerable groups, such as young children, pregnant women or immunocompromised individuals. Therefore, the health risks of a widespread MPXV outbreak are likely higher than what has been observed in recent weeks. Due to its wide species tropism, there is a risk that MPXV might establish animal reservoirs in non-endemic regions, which could fuel future outbreaks. Monitoring of changes in the demography of affected patients and possible spillover events to wildlife or domesticated animals is crucial for risk evaluation and making informed decisions about widening or introducing new safety measures against MPX. Meanwhile, intensifying MPX surveillance and increasing access to vaccines and antiviral drugs can help to diminish the threat of MPX.

## Data Availability

Not applicable.
